# A Multidisciplinary Model for Risk Management and Detection of Ageist Bias in Healthcare Systems in the Era of Artificial Intelligence

**DOI:** 10.3390/healthcare14162642

**Published:** 2026-08-20

**Authors:** Eyal Cohen, Yehuda Adler, Rachel Nissanholtz-Gannot

**Affiliations:** Department of Health Systems Management, Ariel University, Ariel 40700, Israel; yadlercardiol@gmail.com (Y.A.); rachelng@ariel.ac.il (R.N.-G.)

**Keywords:** artificial intelligence ethics, algorithmic bias, clinical decision support systems (CDSS), digital ageism, explainable AI (XAI), medical liability, risk management, bioethics

## Abstract

**Background:** The rapid integration of Artificial Intelligence (AI), specifically Clinical Decision Support Systems (CDSS), into healthcare offers substantial efficiency but introduces critical ethical and legal challenges, particularly the perpetuation of systemic bias against older adults (“Digital Ageism”). While technological advances may improve care, they can violate fundamental bioethical principles when models are trained on unrepresentative data. **Aim:** This article argues that traditional clinical risk-management models are structurally insufficient to address opaque algorithmic bias and presents a conceptual, multidimensional governance framework designed to prevent the codification of human ageism into AI infrastructure. **Methods:** Drawing on systemic failures observed during the COVID-19 pandemic, the normative model integrates legal and governance standards aligned with the EU AI Act, Explainable AI (XAI) tools, and a three-phase implementation protocol. **Results:** To illustrate potential application without overburdening medical staff, the article introduces a theoretical Targeted Escalation Protocol and an Autonomous High-Load Safety Mode. The latter applies deterministic hardcoded constraints to contain age-dominant outputs during acute surges while preserving attending-clinician authority. The framework is explored through an Intensive Care Unit (ICU) thought experiment. **Conclusions:** The framework provides a structured roadmap for policymakers, ethicists, and healthcare administrators to move from reactive defensive medicine toward proactive ethical safety, safeguarding the dignity of the aging population while aiming to mitigate institutional legal exposure.

## 1. Introduction

The digitalization of healthcare systems and the proliferation of Artificial Intelligence (AI) have precipitated a paradigm shift in medical practice. AI in healthcare encompasses a broad spectrum of applications, ranging from diagnostic tools and predictive analytics to administrative algorithms and patient-facing technologies [1]. This article focuses specifically on the evolution of Clinical Decision Support Systems (CDSS). Traditionally, CDSS encompasses a broad spectrum of tools—ranging from simple, rule-based alerts to complex algorithmic frameworks—designed to assist healthcare professionals in diagnostic and therapeutic decision-making across diverse medical domains, including oncology, palliative care, and mental health rehabilitation [2]. It is important to note that while earlier forms of CDSS were not necessarily AI-driven, the contemporary integration of advanced machine learning into these systems introduces a profound ethical tension between operational expediency and the imperative of equitable care.

A central challenge in the deployment of CDSS is the insidious propagation of “bias.” Conceptually, bias in healthcare is a multidimensional construct. In human decision-making, it often manifests as cognitive heuristics or implicit prejudices. In AI systems, however, algorithmic bias emerges when mathematical models systematically and unfairly discriminate against certain individuals or groups, often reflecting and amplifying pre-existing societal inequities [3].

To effectively regulate and mitigate these risks, it is imperative to establish a rigorous conceptual distinction between clinical inaccuracy and systemic algorithmic bias. Clinical inaccuracies occur when a model fails to account for physiological realities—such as misinterpreting age-related changes in bone density or tissue architecture as pathological anomalies rather than normal biological variances. While detrimental, these are technical errors stemming from limitations in clinical precision, governed by traditional medical malpractice frameworks. Conversely, algorithmic bias constitutes a systemic, discriminatory process where the algorithm disproportionately disadvantages a demographic group regardless of physiological ground truths. For instance, systemic exclusion criteria in clinical trials frequently omit older adults with comorbid psychiatric or neurological conditions, resulting in profoundly unrepresentative training datasets [4]. When a CDSS is trained on such skewed data, it commits “representational injustice,” leading to automated discrimination that demands distinct legal and regulatory scrutiny.

One of the most pervasive yet overlooked manifestations of this systemic bias is “Digital Ageism.” Defined as the systemic discrimination against older adults embedded within technological infrastructure, digital ageism encompasses digital neglect, biased datasets, and the outright exclusion of elderly needs in algorithm design [5]. The World Health Organization has explicitly warned against overestimating AI’s benefits without rigorous safeguards against such biases [6].

The urgency of addressing institutional ageism was starkly illuminated during the COVID-19 pandemic. High mortality rates among the elderly were not merely biological inevitabilities but were compounded by institutional triage protocols and resource allocation policies that implicitly devalued older lives [7]. While these pandemic-era failures were largely driven by human judgment and institutional policy rather than AI, they serve as a critical warning. As healthcare transitions to automated triage and predictive analytics, there is a tangible danger that these human biases will become codified into the AI infrastructure, perpetuating ageism at an unprecedented, systemic scale while shielding it from traditional scrutiny.

Compounding the threat of algorithmic bias is the phenomenon of algorithmic opacity, commonly referred to as the “Black Box” problem. The intrinsic complexity of advanced machine learning models prevents clinicians from auditing how a CDSS reached a potentially biased conclusion, thereby neutralizing traditional human oversight. Addressing algorithmic opacity requires a transition from rudimentary human-in-the-loop protocols to comprehensive bias mitigation frameworks. Recent research emphasizes the necessity of robust taxonomies for identifying bias in large-scale models [8] and proposes innovative representation methods for debiasing pre-trained systems [9]. While technical interventions—such as automated self-diagnosis and self-debiasing mechanisms embedded directly into the AI architecture [10]—are essential to reduce the cognitive burden on medical staff, they must be supported by a multidisciplinary governance structure to ensure long-term accountability.

Addressing this opacity requires engagement with foundational scholarship on algorithmic fairness. Barocas, Hardt, and Narayanan show that fairness cannot be reduced to a purely technical correction because mathematical criteria encode normative choices and social structures [11]. XAI also has inherent limits. Explanations can trade fidelity for intelligibility, may fail to capture causality, and can create false reassurance if treated as proof of clinical validity rather than as one element of governance [12].

Therefore, this article argues that traditional clinical risk-management models are insufficient to address the opaque and systemic nature of algorithmic bias in CDSS. We propose a conceptual, multidisciplinary framework that integrates responsibility allocation, Explainable AI (XAI), and medical risk management. By establishing a structured three-phase pathway spanning procurement auditing, clinical deployment, and post-market surveillance, the model offers a conceptual roadmap intended to safeguard the dignity and autonomy of older adults while aiming to mitigate institutional legal exposure.

### 1.1. The Collapse of Traditional Models

Healthcare systems currently rely on three main risk management models: Reactive (investigating events after they occur) [13]; Interactive (real-time collaboration) [14]; and Proactive (identifying risk factors in advance) [15]. The proactive model relies mainly on Comprehensive Geriatric Assessment (CGA) [16], a multidisciplinary process for assessing the patient’s physical, mental, and social status. Additional tools include the PACE program and the World Health Organization’s ICOPE framework [17,18].

Despite their importance, these models failed during the COVID-19 pandemic. They proved “blind” to ageist bias as a systemic risk source. High mortality among the elderly stemmed not only from biological vulnerability but also from failed risk management fueled by ageism. In the first wave, approximately one-third of the deceased in Israel were residents of geriatric institutions (who comprise only 3% of the elderly population), a disaster stemming from failures in supplying protective equipment and poor preparedness [19]. Furthermore, strict restrictions such as “confinement” in rooms and prohibition of visits led to severe loneliness and death in isolation. The care of the elderly was tainted by prejudices that worsened during the pandemic and became lethal.

While this statistical evidence is drawn from the localized Israeli experience, it serves as a critical proxy for a universal vulnerability: human ageist heuristics escalate during crises of resource scarcity. As healthcare transitions to AI, the danger is that these human-mediated biases will be codified into algorithmic triage.

This heavy mortality should have served as a “Red Flag,” mandating a re-examination of existing models. The failure of traditional models stemmed primarily from the lack of attention to ageism as a central risk factor—a failure that led to the exclusion of the elderly from society, deterioration in their health and mental state, and increased mortality, particularly in geriatric institutions where heavy mortality was recorded due to preparedness failures [7].

### 1.2. The Intensifying Threat: Digital Ageism

Artificial Intelligence (AI) holds tremendous promises for medicine and healthcare, yet the World Health Organization warns against overestimating its benefits, highlighting risks such as algorithmic bias and privacy violations [6]. The central concern is that algorithms reflect social biases and birth “Digital Ageism,” a phenomenon manifested in the neglect of older adults’ needs in technology development, biases in datasets, and digital inaccessibility [5]. While the fear of perpetuating racism and sexism through AI has garnered attention, the issue of ageism has not yet been deeply explored in the field of risk management.

Digital ageism exacerbates risks in the AI era, as it includes digital neglect, algorithmic biases, and inaccessibility that limit elderly participation in health services. This leads to the perpetuation of discrimination through a “Black Box” where algorithmic processes are not transparent, making it difficult to identify embedded ageist biases, thus endangering the health and well-being of the elderly.

A concrete manifestation of proxy-based inequity in existing healthcare AI is the use of historical healthcare costs as a proxy for health needs, a practice shown to encode prior disparities into risk scores [3]. That evidence was established in relation to racial disparities, not age-specific discrimination. Its relevance to Digital Ageism is therefore a reasoned extension: older adults with complex needs may also be mischaracterized when expenditure patterns are treated as equivalent to clinical need, but this age-specific pathway requires direct empirical validation [5].

The primary aim of this theoretical study is to develop a comprehensive, multidisciplinary governance framework capable of detecting and mitigating ageist bias in clinical AI systems. Specifically, the objectives of this paper are:

1. To outline a three-phase operational pathway integrating procurement auditing, clinical deployment, and post-market surveillance.
2. To propose a tripartite governance and responsibility model designed to operationalize relevant EU AI Act duties without displacing applicable civil-liability rules.
3. To introduce a Targeted Escalation Protocol, including an Autonomous High-Load Safety Mode, designed to balance algorithmic oversight with the operational realities of acute and high-volume care.

## 2. Principles of the Multidimensional Model: A Three-Phase Implementation Framework

This article proposes a conceptual, multidisciplinary risk-management model in response to these detection failures. The model complements existing reactive, interactive, and proactive approaches by treating ageist bias as a lifecycle safety risk. It combines pre-market auditing, clinical deployment controls, autonomous deterministic safeguards for acute or high-load conditions, and continuous post-market surveillance. The model does not assume that every decision will receive real-time committee review. Instead, it assigns routine governance and calibration to multidisciplinary teams while permitting a predefined safety layer to act immediately when operational delay would itself create risk.

### 2.1. Phase I: Procurement and Vendor Auditing (Identification of Algorithmic “Red Flags”) [15]

Before adopting an AI diagnostic tool, healthcare institutions should require provider-side evidence concerning dataset representativeness, subgroup performance, documentation, and foreseeable bias risks. Where contractual access and technical capacity permit, institutional review may supplement the provider audit. These measures are intended to identify potential age-related risks before deployment and to support proportionate corrective action, rather than to guarantee that all clinical errors will be prevented.

Furthermore, while standard medical AI supply chains involve hospitals procuring “frozen” commercial models rather than actively managing decentralized training networks, the procurement phase must demand developer-side audits. Hospitals should require vendors to use or evaluate Federated Learning (FL) methodologies during pre-market development where appropriate. FL can enable collaborative training across decentralized data consortia, enhance demographic diversity, and reduce direct sharing of patient records, thereby placing the primary burden of representational justice on the provider rather than the purchasing hospital [20].

However, FL is not a panacea. It introduces technical and governance challenges, including data heterogeneity, communication overhead, privacy and security risks, and the possibility of perpetuating bias when participating institutions share similar exclusionary practices [20].

When geriatric representational gaps are extensive, human oversight cannot compensate for a fundamentally skewed evidentiary base. Procurement should therefore pause or restrict deployment until adequate real-world geriatric data are collected. Synthetic augmentation and federated fine-tuning may be considered only as supplementary techniques after a targeted multi-center empirical seed exists. Synthetic data cannot reliably invent non-existent physiological realities and should not be used to conceal an absence of representative source data.

### 2.2. Phase II: Clinical Deployment and Human-in-the-Loop Protocols (Multidisciplinary Control Teams) [21]

During active clinical use, multidisciplinary teams including experts in medicine, gerontology, sociology, ethics, and law govern threshold calibration, periodic audits, and escalated non-acute cases [21]. They are not expected to review every CDSS output. In acute emergencies or high-load conditions, the Autonomous High-Load Safety Mode applies a deterministic hardcoded constraint without waiting for the team, while the attending clinician retains final authority over irreversible treatment and access-to-care decisions. The MDT then reviews the logged event retrospectively, identifies systemic patterns, and refines institutional protocols.

Integration of Autonomy and Human Dignity Principles: The deployment phase will also embed overarching principles for protecting human sovereignty over one’s body. The goal is to protect the autonomy of individuals with disabilities or vulnerabilities [22], allowing them to make independent decisions, make mistakes, and learn from them, avoiding the appointment of a guardian if less restrictive alternatives exist. The model will verify that AI systems and medical procedures support the elder’s autonomy through protocols ensuring algorithmic decisions consider the elder’s will [23].

### 2.3. Phase III: Continuous Post-Market Surveillance (Regulatory and Ethical Framework)

The model proposes organizational procedures for fair and equitable AI use, transparency, and digital accessibility. These procedures should be informed by WHO guidance [6,23,24], applicable regulation, and local evidence. Continuous post-market surveillance is intended to support accessibility, detect emerging subgroup disparities, and enable administrative and public oversight, but its effectiveness depends on institutional capacity and enforcement [25].

To support autonomy and human dignity, the framework proposes Participatory Design (co-creation). Older adults should be involved in CDSS development, usability testing, and the local calibration of Bias Sensitivity Thresholds. Treating older patients as active stakeholders may reduce paternalism and digital neglect, but participatory processes do not by themselves eliminate structural bias and must be evaluated in practice.

**Figure 1:**
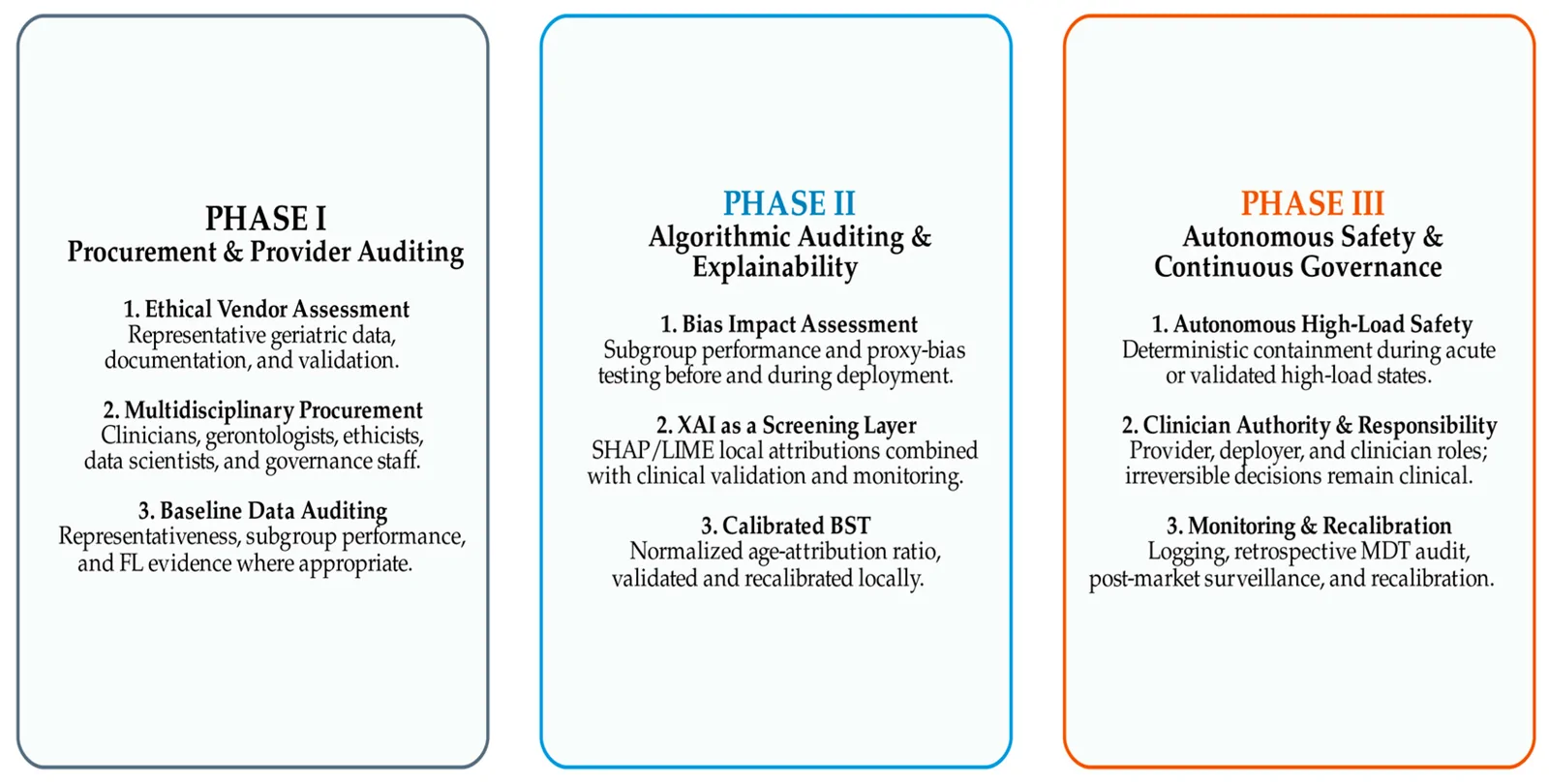
Three-phase multidisciplinary framework with autonomous high-load safety and lifecycle governance.

The multidimensional model is designed to supplement reactive, interactive, and proactive approaches by adding lifecycle auditing, calibrated escalation, autonomous high-load containment, and post-market learning. Transparent analysis may help identify embedded age-related risks in datasets, code, and applications such as medical imaging, but it cannot guarantee that bias will be detected or removed. Prospective validation and subgroup monitoring remain essential (Figure 1).

## 3. Economic and Legal Implications: Clarifying Liability

A foundational pillar of the proposed model is a conceptual allocation of functional responsibility within a complex digital ecosystem. Ambiguity over who must audit, configure, oversee, and respond to AI outputs can encourage defensive practices and economic waste [26,27]. The framework therefore maps provider, deployer, and clinician responsibilities in a manner intended to complement, rather than replace, obligations under the EU Artificial Intelligence Act and applicable national law [28].

### 3.1. The Tripartite Liability Framework

It is crucial to distinguish between legally binding obligations imposed by the EU AI Act and the governance extensions proposed in this article. The tripartite allocation below is a conceptual model for operationalizing regulatory duties, not a comprehensive civil-liability doctrine. Applicable product-liability, medical-malpractice, contractual, and national rules continue to govern legal outcomes. The model therefore identifies functional domains of responsibility without claiming that the EU AI Act itself assigns all resulting civil liability [28].

**Figure 2:**
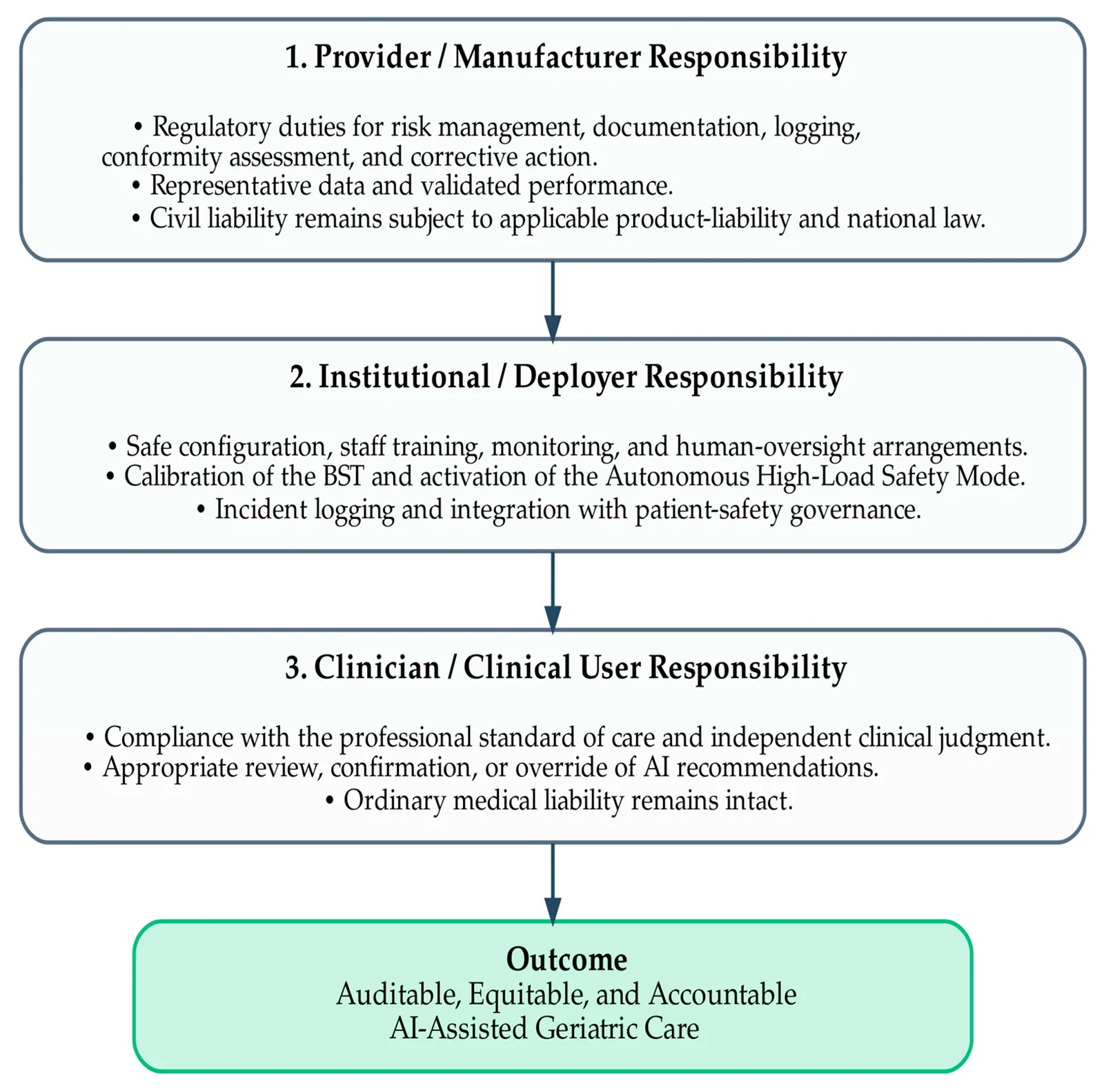
Liability and regulatory responsibility map.

To reduce ambiguity, the model proposes a tripartite allocation of functional responsibility among providers, deployers, and clinicians (see Figure 2). The allocation is a governance tool and does not guarantee complete or exclusive attribution in an individual dispute:

1. **Provider/Manufacturer Regulatory Responsibility:** The EU AI Act imposes regulatory obligations on providers of high-risk AI systems, including compliance with the applicable Chapter III requirements, quality management, documentation, logging, conformity assessment, and corrective action, particularly through Article 16 and related provisions [28]. These duties do not by themselves establish strict civil liability for every adverse outcome. Under the proposed governance model, provider responsibility concerns failures attributable to design, documentation, validation, monitoring, or non-representative data, subject to applicable product-liability and national law.
2. **Institutional/Deployer Governance Responsibility:** Article 26 requires deployers of high-risk AI systems to take appropriate technical and organizational measures, assign competent human oversight, monitor operation, and, where they control input data, ensure that those data are relevant and sufficiently representative [28]. The proposed model therefore assigns institutions governance responsibility for configuration, staff training, threshold calibration, incident logging, and implementation of autonomous safety safeguards.
3. **Professional Medical Responsibility (The Clinician):** The model adds a specific focus on automation bias but does not narrow ordinary medical liability. Clinicians remain bound by the professional standard of care and independent clinical judgment. Article 14 requires high-risk systems to be designed so that they can be effectively overseen by natural persons; the precise duties of clinicians and institutions continue to depend on applicable medical and national law [28].

By creating a transparent and auditable trail of algorithmic, clinical, and institutional judgment, the model may facilitate earlier regulatory learning and more accurate attribution of responsibility. It cannot eliminate litigation or predetermine liability.

Distributing responsibility across three actors also creates a risk of responsibility diffusion. Effective implementation therefore requires clear contractual demarcation, named audit ownership, incident-escalation rules, and integration with hospital patient-safety committees to support the resolution of accountability disputes.

### 3.2. Operational Feasibility, Economics, and Autonomous Safety

A central practical challenge for multidisciplinary governance is the risk of operational delay and economic burden, particularly in acute or high-volume environments. Requiring an ethics and legal committee to review routine CDSS outputs would be clinically unworkable. The proposed framework therefore separates real-time algorithmic containment from periodic multidisciplinary governance.

Technical self-diagnosis and self-debiasing may reduce the cognitive burden on medical staff, but evidence from natural-language processing [10] illustrates a technical direction rather than clinical validation. The framework therefore treats automated mitigation as one safety layer that must be prospectively tested, monitored, and combined with human clinical authority.

Consequently, the Human-in-the-Loop (HITL) multidisciplinary team is not a constant fixture for every algorithmic decision. In non-acute cases, the team functions as a specialized escalation tier when a case exceeds the calibrated BST. In acute emergencies or high-load states, the system does not wait for synchronous MDT review; it activates the autonomous deterministic safety constraint described below and places the event in a retrospective review queue.

During acute surges, the Autonomous High-Load Safety Mode operates at the software safety layer. A high-load state is activated automatically when pre-specified, locally validated operational indicators exceed institutional thresholds, for example sustained ICU occupancy, triage queue length, clinician-to-patient ratio, or decision latency. While active, the mode continuously evaluates the normalized age-attribution ratio, applies locally calibrated constraints, suppresses or quarantines an age-dominant output, and generates a conservative fallback based on validated physiological variables. It autonomously contains the algorithmic recommendation but does not replace the treating clinician: it may not independently initiate irreversible treatment or deny access to care. The mode deactivates when validated recovery criteria are met, and every activation, intervention, override, and deactivation is logged for retrospective MDT review and post-market surveillance. Repeated borderline cases trigger institution-level recalibration of the BST rather than repeated interruption of daily emergency operations.

From a health-economics perspective, maintaining governance, audit infrastructure, and an on-call escalation pathway requires resources. Those costs may be offset by earlier detection of systemic failures, reduced regulatory exposure, and avoidance of repeated harmful deployment, but this proposition requires dedicated economic evaluation. The framework should therefore be understood as a candidate for pilot implementation and prospective validation, not as an already established scalable standard of care.

## 4. Illustrative Thought Experiment: Applying the Multidisciplinary Model

Rather than presenting an empirical validation, which is beyond the scope of this conceptual paper, this section presents an illustrative thought experiment. This theoretical scenario explores the potential application of the proposed framework within a hypothetical Intensive Care Unit (ICU) triage environment, demonstrating how the normative principles discussed above could function in a high-stakes clinical setting.

### 4.1. Simulation Scenario: Acute Triage and the “Resource Allocation” Algorithm

**The Clinical Context:** A 79-year-old patient, presenting with acute respiratory distress syndrome (ARDS), requires an immediate decision regarding ICU admission and mechanical ventilation. The hospital utilizes a CDSS model designed to predict “Survival Probability and Resource Utility” to assist in triage during periods of high occupancy.

**The Algorithmic Failure:** The CDSS generates a “Low Priority” recommendation for the patient. Unlike routine clinical errors, this recommendation is driven by **Representational Injustice**. The underlying training dataset lacked age-stratified physiological data for septic shock in octogenarians, causing the algorithm to rely on “Age” as a proxy for fragility, rather than on the patient’s actual functional vitality. This is a classic manifestation of **Digital Ageism**.

### 4.2. Operationalizing Phase II: XAI and Technical Metrics

Under the proposed model, the “Low Priority” flag first triggers an automated bias screen. In a non-acute case, an elevated BST activates remote MDT review. In an acute or high-load case, the Autonomous High-Load Safety Mode applies the deterministic constraint immediately, while preserving attending-clinician authority. Instead of treating the CDSS as an unquestioned “Black Box,” the system uses XAI outputs as one auditable signal within the broader governance process.

1. **Explainability Tools (SHAP/LIME):** The system generates local feature-attribution outputs. In this illustrative example, age-related proxy variables contribute the largest local attribution to the negative recommendation, overshadowing positive indicators such as baseline functional status and stable renal function. The example does not treat SHAP values as global percentages; the age contribution is evaluated using the normalized attribution ratio defined below [29,30].
2. **Threshold Metrics:** The institution defines a Bias Sensitivity Threshold (BST). Since SHAP values represent local feature attributions relative to a base expected value rather than absolute global percentages, the system calculates the relative magnitude of the age feature. If the ratio of the absolute SHAP value for the age proxy (|*φ_age_*|) to the sum of all absolute feature attributions (Σ_i=1_^M^|*φ_i_*|) exceeds the pre-defined *τ_BST_* in a life-critical recommendation, the system classifies the output as “High Risk for Bias.”
3. It is important to distinguish between the components of this workflow. SHAP and LIME are established computational techniques for local explanation [29,30], although their clinical reliability remains limited [12]. By contrast, the BST, Targeted Escalation Protocol, and Autonomous High-Load Safety Mode are novel normative constructs introduced in this framework. The numerical value of the BST must not be arbitrary; it should be calibrated through expert consensus, retrospective simulation on local hospital data, prospective pilot testing, and evolving regulatory guidance.

#### 4.2.1. Algorithmic Representation of the Targeted Escalation Protocol

To illustrate the model in a high-paced Emergency Room (ER) or ICU setting, the following Python pseudocode in Algorithm 1 presents the core logic of the Bias Sensitivity Threshold (BST). It distinguishes non-acute asynchronous MDT review from acute or high-load autonomous containment, uses holistic patient and operational data, and preserves attending-clinician authority over final care decisions.
**Algorithm 1:** Targeted Bias-Aware Triage Escalation and Autonomous High-Load MitigationInput: Patient Electronic Medical Record (EMR) data; CDSS prediction model; XAI engine; institutional BST; operational-load indicator.Output: Clinician-validated decision, audit record, and post-market surveillance event.1. Data Acquisition: Extract clinical status, social factors, age, and the current operational-load state from the EMR and hospital systems.2. Primary Inference: Generate the baseline triage recommendation through the CDSS.3. XAI Decomposition: Use SHAP or LIME to quantify the local importance of age and age-proxy variables.**4. Threshold Gatekeeper: Compute the normalized age-attribution ratio:**   If *R_age_* ≥ *τ_BST_*, where *R_age_* = |*φ_age_*|/Σ_i=1_^M^|*φ_i_*|    a. Log the threshold event and preserve the explanation output.    b. Determine whether the case is acute or whether pre-specified institutional load indicators exceed the locally validated activation threshold.    c. If Acute/High-Load: automatically activate the Autonomous High-Load Safety Mode; suppress or quarantine the age-dominant output; generate a conservative fallback from validated physiological variables; notify the attending clinician for immediate confirmation or override; maintain the mode while the activation threshold remains exceeded; then deactivate under validated recovery criteria and queue retrospective MDT review.    d. If Non-Acute: trigger remote MDT review and Comprehensive Geriatric Assessment; the MDT validates or adjusts the recommendation before final clinician approval.5. Else (Standard Risk): proceed to routine physician validation.6. Termination: return the clinician-validated decision and archive the event for post-market surveillance.

#### 4.2.2. Emergency and High-Load Protocol: Autonomous Deterministic Safety Constraint

To address operational latency in acute emergency settings and periods of sustained clinical overload, the framework introduces an Autonomous High-Load Safety Mode implemented through a deterministic hardcoded safety constraint. The mechanism activates automatically when an acute-case flag or a locally validated operational-load threshold is crossed, acts without waiting for synchronous MDT review, and deactivates automatically when validated recovery criteria are satisfied.

The mechanism is not a learning or generative decision-maker and does not formulate new ethical or legal rules. It autonomously applies pre-specified, locally validated constraints derived from clinical governance and applicable regulatory requirements. Let *w_age_* denote the normalized local attribution ratio defined above. The safety condition is:

*w_age_* ≤ *τ_anchor_* (1)

We acknowledge that this inequality is a conceptual abstraction rather than a complete mathematical representation of clinical reality. Chronological age rarely acts independently from physiological frailty, comorbidity burden, functional reserve, and interactions among model features.

If *w_age_* exceeds the locally calibrated *τ_anchor_*, the mechanism suppresses or quarantines the age-dominant output and produces a conservative fallback recommendation from validated non-age clinical variables. It cannot independently authorize an irreversible intervention or deny care. The attending physician receives the fallback together with an audit trail and retains authority to confirm or override it; the event is queued for retrospective MDT review.

This formalization is intended to reduce the probability that digital ageism propagates during time-critical care. It does not guarantee bias elimination and requires prospective validation, fail-safe testing, cybersecurity safeguards, and local threshold calibration.

### 4.3. Multidisciplinary Intervention and Regulatory Compliance

Following the instantaneous autonomous intervention by the deterministic safety constraint, the attending physician receives the fallback recommendation and audit information without waiting for a committee. The multidisciplinary team, comprising a senior intensivist, a gerontologist, and legal or governance counsel, convenes retrospectively in acute cases, or asynchronously before completion in non-acute cases, to evaluate the logged “Red Flag” and refine institutional protocols.

1. **Clinical Realignment:** In the immediate case, the attending intensivist evaluates the patient-specific physiological data and confirms whether the conservative fallback is clinically appropriate. Retrospective gerontological review then assesses whether chronological age was improperly used as a proxy for frailty.
2. **Legal and Regulatory Framework:** Where the CDSS qualifies as a high-risk AI system under the applicable classification rules, the institution must implement the human-oversight arrangements specified for the system and maintain appropriate logs [28]. The retrospective review documents governance and supports corrective action; it does not automatically shield the institution or clinician from liability.

**Figure 3:**
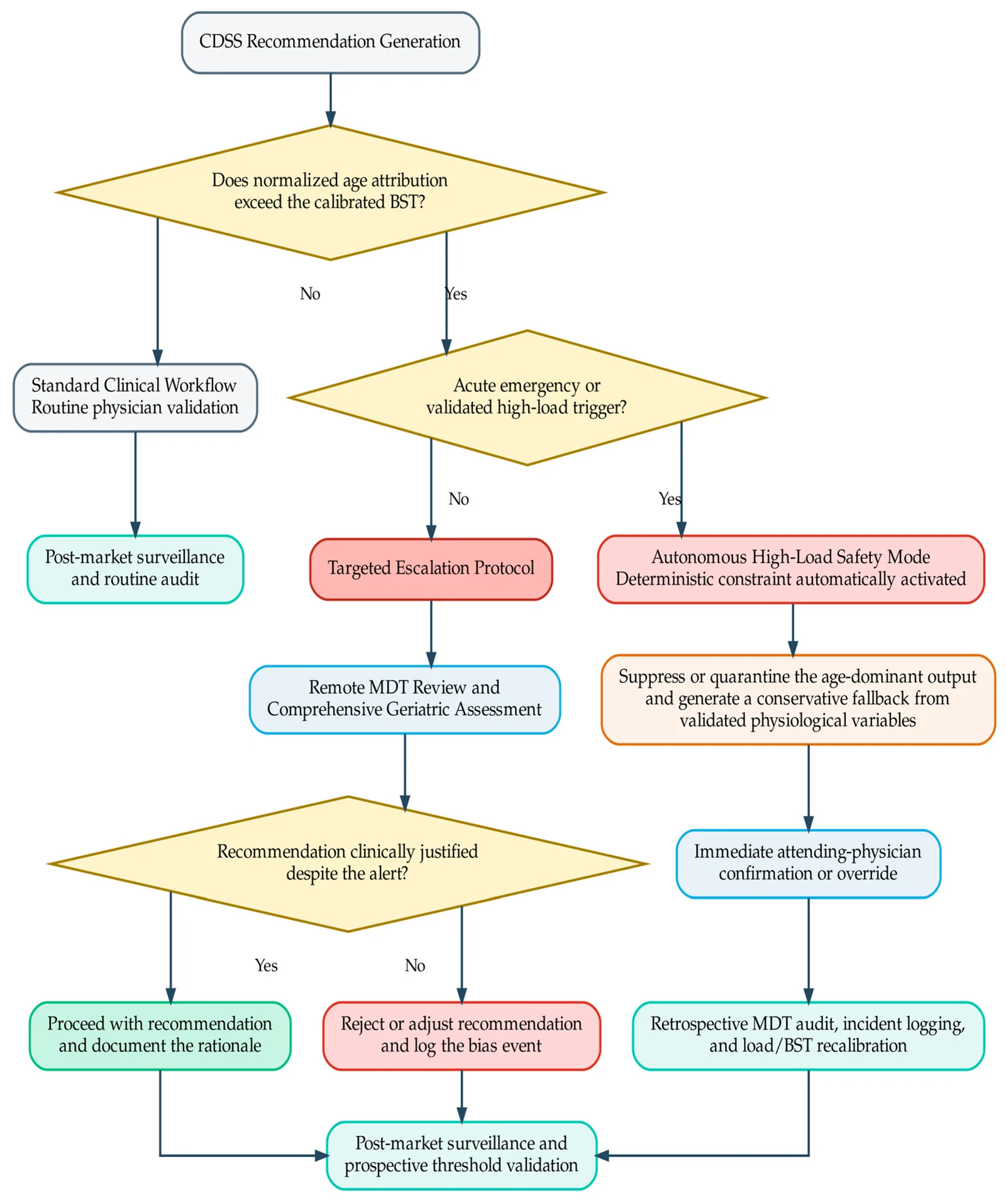
Standard and autonomous high-load ICU governance workflow.

The Result: The Autonomous High-Load Safety Mode suppresses the age-dominant “Low Priority” output and presents a conservative fallback for immediate physician review. In this thought experiment, the attending intensivist confirms ICU admission and the patient recovers. The MDT later reviews the log and recalibrates institutional safeguards if necessary. Figure 3 visualizes both the non-acute escalation pathway and the autonomous acute or high-load pathway. The scenario illustrates potential operation of the framework; it is not empirical proof of clinical effectiveness.

## 5. Discussion

This study introduces a multidimensional governance framework aimed at mitigating Digital Ageism in AI-driven healthcare systems. Traditional patient-safety models remain indispensable, but they tend to organize responsibility around clinical events, institutional processes, or post-incident learning [13,14,15]. The proposed framework adds a lifecycle perspective: representational risk is examined during procurement, monitored during deployment, and revisited through post-market surveillance. It is intended to complement rather than replace existing clinical risk-management structures.

The model also treats algorithmic fairness as a sociotechnical problem rather than a single mathematical optimization task. Barocas, Hardt, and Narayanan emphasize that fairness criteria embody normative choices and cannot by themselves resolve structural inequality [11]. Consistent with that position, the BST is not presented as a universal moral boundary. It is a locally calibrated governance trigger that identifies when age or age proxies may be exerting disproportionate influence and when additional safeguards are warranted.

A related limitation concerns explainability. SHAP and LIME can provide useful local attribution information [29,30], but current XAI techniques do not necessarily reveal causal reasoning or guarantee clinical validity [12]. Accordingly, this framework uses XAI as a screening and audit layer, not as proof that a recommendation is fair. The normalized attribution ratio can generate false positives or false negatives, and every implementation requires external validation, subgroup testing, and monitoring for distribution shift.

The COVID-19 experience in Israel is therefore used as an illustrative warning rather than as universal empirical validation. Similarly, the healthcare-cost proxy example demonstrates how historical institutional patterns can be encoded into a prediction model [3], but its extension to age-specific discrimination remains a reasoned hypothesis that requires direct testing in older populations [5]. This distinction is important because the framework should not convert an analogy into an unsupported causal claim.

Operational feasibility is addressed through a dual pathway. In non-acute cases, Targeted Escalation permits multidisciplinary review before final approval. In acute emergencies or sustained high-load conditions, the Autonomous High-Load Safety Mode performs immediate deterministic containment at the software layer. It can suppress or quarantine an age-dominant output, generate a conservative fallback, and create an audit record without waiting for the MDT. Autonomy is therefore assigned to the safety mechanism, not to an independent clinical decision-maker. Irreversible treatment and access-to-care decisions remain under attending-clinician authority, while the MDT reviews events retrospectively and recalibrates the system.

The legal framework must likewise remain cautious. The EU AI Act establishes regulatory duties for providers and deployers of high-risk systems, including requirements relating to risk management, documentation, monitoring, and human oversight [28]. It does not itself supply a complete tripartite civil-liability code. The allocation proposed here is therefore a governance map that should be implemented through contracts, patient-safety committees, professional standards, and applicable national law. This approach also acknowledges the risk of responsibility diffusion and the need for clear escalation and audit ownership.

Finally, the framework remains a normative proposal. Its clinical value, economic feasibility, and effect on alert burden must be tested prospectively across diverse institutions, including resource-constrained settings. Table 1 summarizes the principal differences between traditional risk management and the proposed AI-governance model, while the limitations below identify the empirical work required before broad adoption can be justified.

## 6. Summary of Application: Toward a Paradigm Shift in Geriatric Risk Management

The proposed multidimensional model is designed to supplement traditional risk management by addressing the sociotechnical gap in AI-driven healthcare. Phase I focuses on provider-side auditing and representative development data; Phase II combines calibrated escalation with autonomous deterministic containment during acute or high-load conditions; and Phase III supports continuous surveillance and institutional learning. The framework treats algorithmic bias as a potential safety risk rather than a purely technical anomaly. Its contribution is an auditable governance pathway that may help institutions align practice with transparency, monitoring, and human-oversight requirements while protecting geriatric dignity. Its effectiveness remains to be established through pilot implementation and empirical evaluation.

## 7. Limitations and Suggestions for Future Research

Several limitations must be acknowledged. First, as a theoretical model, the framework remains contingent on data quality. Even rigorous MDT oversight cannot compensate for fundamentally skewed datasets when representational gaps are extensive. The framework has not yet been prospectively validated, and longitudinal pilot studies are required to establish clinical effectiveness, false-positive and false-negative rates, and effects on patient outcomes.

Operational and distributive constraints are equally important. The economic feasibility of governance infrastructure and an on-call Targeted Escalation tier remains unproven, particularly in resource-constrained institutions and low- and middle-income countries. Implementation also depends on staff algorithmic literacy, cybersecurity capacity, and local calibration. Global use requires evaluation of statistical heterogeneity across regions and direct study of intersectional bias involving age, race, gender, disability, and socioeconomic status.

The Autonomous High-Load Safety Mode introduces additional risks, including inappropriate suppression of clinically valid outputs, false reassurance from fallback recommendations, cybersecurity failure, overreliance by fatigued clinicians, and miscalibration of activation or recovery thresholds. Before deployment, it requires prospective fail-safe and override testing, local workflow simulation, validation of load indicators, monitoring of mode switching, and continuous review of differential performance across age and intersectional groups. It is not a substitute for clinical judgment.

## 8. Conclusions

The digitalization of geriatric care offers substantial clinical promise but also creates a risk that existing ageist assumptions will be embedded in automated systems. The failures observed during the COVID-19 pandemic provide a warning about how scarcity and institutional heuristics can disadvantage older adults, but they do not by themselves validate the proposed framework. This article therefore presents a conceptual starting point for lifecycle governance: provider-side auditing, calibrated Bias Sensitivity Thresholds, targeted multidisciplinary escalation, an Autonomous High-Load Safety Mode for immediate software-layer containment, and continuous surveillance. The autonomous mechanism is designed to function during acute surges without waiting for committee review, while preserving attending-clinician authority over irreversible treatment and access-to-care decisions. The framework aims to mitigate algorithmic ageism and improve accountability, but broad applicability requires pilot implementation, prospective empirical validation, local calibration, and evaluation across diverse and resource-constrained healthcare systems.

## Figures and Tables

**Table 1:** Comparison of Traditional Clinical Risk Management vs. The Proposed Multidisciplinary Model.

Feature	Traditional Clinical Risk Management	Proposed Multidisciplinary AI-Governance Model
**Focus**	Reactive (Mitigating harm post-incident)	Lifecycle-oriented (identifying and mitigating algorithmic harm before, during, and after deployment)
**Primary Actor**	Clinician/Hospital administration	Multidisciplinary and lifecycle-based (providers/developers, institutions, clinicians, ethicists, and patient-safety teams)
**Transparency**	Relies on clinical documentation; algorithmic processes remain an opaque ‘Black Box’	Uses XAI as one auditable screening layer, supplemented by clinical validation and human oversight
**Liability**	Concentrated primarily on the clinician (Malpractice) and the institution, but struggles to attribute liability for opaque ‘Black Box’ algorithmic failures.	Conceptually allocated among the provider, institution, and clinician, subject to applicable law
**Bias Mitigation**	Ad hoc; heavily dependent on individual physician awareness and intuition	Systemic and auditable; uses calibrated BSTs and autonomous deterministic safeguards
**Ethical Goal**	Non-maleficence (Do no harm)	Algorithmic equity and mitigation of ‘Digital Ageism’

## Data Availability

No new data were created or analyzed in this study. Data sharing is not applicable to this article.
